# A Modular Design for Distributed Measurement of Human–Robot Interaction Forces in Wearable Devices

**DOI:** 10.3390/s21041445

**Published:** 2021-02-19

**Authors:** Keya Ghonasgi, Saad N. Yousaf, Paria Esmatloo, Ashish D. Deshpande

**Affiliations:** Rehabilitation and Neuromuscular Robotics Lab, The University of Texas at Austin, Austin, TX 78712, USA; keya.ghonasgi@utexas.edu (K.G.); yousaf@utexas.edu (S.N.Y.); p.esmatloo@utexas.edu (P.E.)

**Keywords:** physical human robot interaction, exoskeleton attachment design, force sensing

## Abstract

Measurement of interaction forces distributed across the attachment interface in wearable devices is critical for understanding ergonomic physical human–robot interaction (pHRI). The main challenges in sensorization of pHRI interfaces are (i) capturing the fine nature of force transmission from compliant human tissue onto rigid surfaces in the wearable device and (ii) utilizing a low-cost and easily implementable design that can be adapted for a variety of human interfaces. This paper addresses both challenges and presents a modular sensing panel that uses force-sensing resistors (FSRs) combined with robust electrical and mechanical integration principles that result in a reliable solution for distributed load measurement. The design is demonstrated through an upper-arm cuff, which uses 24 sensing panels, in conjunction with the Harmony exoskeleton. Validation of the design with controlled loading of the sensorized cuff proves the viability of FSRs in an interface sensing solution. Preliminary experiments with a human subject highlight the value of distributed interface force measurement in recognizing the factors that influence ergonomic pHRI and elucidating their effects. The modular design and low cost of the sensing panel lend themselves to extension of this approach for studying ergonomics in a variety of wearable applications with the goal of achieving safe, comfortable, and effective human–robot interaction.

## 1. Introduction

Applications for wearable robots are emerging in many fields such as rehabilitation, assistive devices, human augmentation, and haptics [1,2,3]. Safety and comfort are major factors in the use of such robots, especially with sensitive populations where these aspects correspond to the high rate of disuse of assistive devices [4]. Both safety and comfort are directly affected by the robot’s control and its manifestation across physical human–robot interaction (pHRI) interfaces, consisting of the mechanical attachment between the user and the robot [5]. These interfaces range from fully compliant cloth straps to rigid splints that must be worn by the user, resulting in vastly different interaction effects. The magnitude and distribution of pressures at the interface characterize the interaction, where undesired interaction effects may result in inefficient performance and, in the worst case, lead to injury [4].

Prior research on pHRI has studied this interaction through the use of torque sensors at robot joints or six-axis load cells at attachments between the interface and the robot (Harmony [6], ABLE [7], and ALEX II [8]). However, both of these methods are insufficient to fully capture the distributed nature of interaction at the interface. Specifically, these methods need accurate models of the human–robot system, must be robust to changes in the system dynamics, and are limited by only having force/torque measurements from a single point. On the other hand, measurement of distributed interaction effects across the interface provides a more complete picture, but the studied solutions have been either expensive [9] or unreliable [10]. The success of distributed load sensing at the interface depends on the ability to measure fine effects of the interaction at the skin surface of the wearer, but sensing at this level has posed several challenges.

Opto-electronic transducers [9,11] have been used to create a sensing solution with soft silicone covers to measure the force between the robot and the user interface. The works propose utilizing arrays of these sensors at the attachment points and have tested their technology on an upper-limb exoskeleton (NEUROExos) [12], a lower-limb exoskeleton (LOPES) [11], and a foot insole for monitoring gait behavior [13]. Despite the high accuracy and repeatability of measurements by opto-electronic sensors, the technology and manufacturing process are expensive and not suitable for low-cost prototyping. For instance, the silicone covers developed in [9,11] must be custom-made for each application, and the second generation of sensors requires flexible printed circuit boards (PCBs) [14]. Moreover, opto-electronic transducers are not available as commercial off-the-shelf products that can be easily implemented into a distributed sensing solution.

Force-sensing resistors (FSRs) offer a more accessible approach to distributed load sensing due to their low cost, off-the-shelf availability, and versatile low-profile form factor. These sensors use piezoresistive technology to relate applied forces to a change in material resistance. In the context of pHRI applications, Tamez-Duque et al. [15] used FSRs to measure strap tension in a lower-body exoskeleton towards avoiding discomfort, pain, and injuries. Rathore et al. [16] utilized FSRs to measure peak forces experienced in the lower-limb REX exoskeleton. Bessler et al. [10] implemented FSRs for interaction sensing in conjunction with a force/torque sensor to measure forces that occur between an arm and a splint. The authors [10] concluded that readings from FSRs are not reliable or robust, as most of the sensors did not show any force values during the experiment. These results further emphasize the challenge of capturing forces resulting from the interaction of compliant human skin and a rigid robot interface.

Implementing FSRs to be compatible with the wearable robot and the human wearer while simultaneously following the guidelines set forth by manufacturers [17] is not trivial. Specifically, FSRs require a rigid interface called a puck to load the forces applied to the FSR surface within its sensing region. However, the human skin surface, where interaction occurs, is neither rigid nor consistent in stiffness properties during dynamic loading. This mismatch is a potential explanation for the inconsistent results in [10], where the arm was directly interfaced with FSRs. Further, a good sensing solution is expected to be used with a variety of interface designs such as cuffs, prostheses, or splints. A complicated implementation of the sensing solution would make such customizations intractable.

In this paper, we address these challenges by focusing on integration and modularity of a sensing solution using FSR technology. In Section 2, we propose a single sensing panel that ensures rigid force distribution at the surface of the FSR through a puck design, decoupling of loads from adjacent panels, and ergonomic attachment to the human via padded curvatures that allows for force transmission through the sensors while ensuring comfortable contact. The modular design of the panel lends itself to exploring a variety of research questions regarding distributed interaction forces while making minimal changes to the sensing hardware. In this paper, modularity refers to how a single panel is treated as a standard sensing unit that can be repeated in an array of sensors across a pHRI interface to capture distributed loads. The instrumentation can be scaled as necessary depending on the overall size of the pHRI interface and which areas need to be sensorized. In this context, scalability of the sensing solution refers to how easily it can be adapted for different applications.

**Figure 1:**
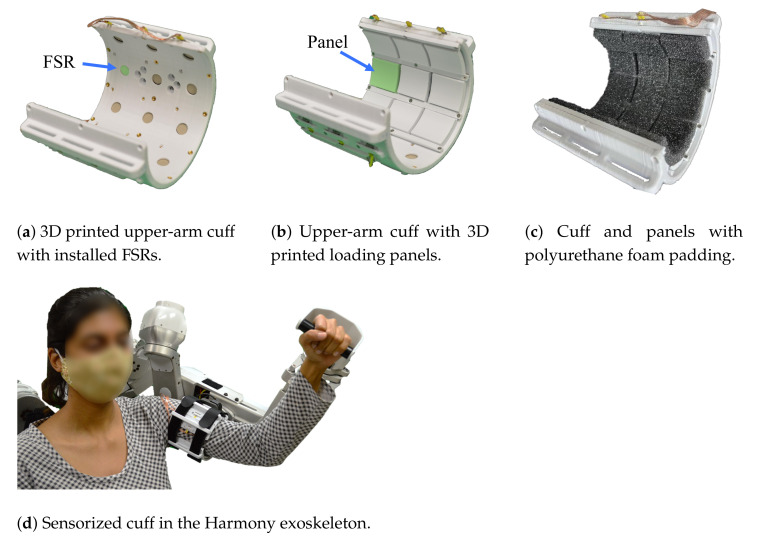
The design introduced in this work is implemented for an upper-arm exoskeleton interface. The upper-arm cuff uses 24 panels, each covered with foam padding, to allow for comfortable interaction between the wearer and the interface.

We demonstrate the use of the sensing panel design through an upper-arm cuff attachment which uses 24 panels (Figure 1a–c). The performance of the sensing panel is studied in Section 3, validating the use of an array of sensors and the cuff design through rigorous experiments. The observed agreements in trends between the measured distributed loads and the ground truth measurements from a force/torque sensor are crucial to develop confidence in the reliability and repeatability of FSR force measurements. In Section 4, we present three potential applications of the sensing panel in conjunction with the upper-body Harmony exoskeleton [6] (Figure 1d). The three case studies investigate (i) the effects of cuff strap tightness on the distributed loads on the arm, (ii) the distributed loads on the arm during an active (voluntary) and passive (robot-assisted) flexion of the elbow, and (iii) the effects of different padding stiffness on the distribution of the loads on the human arm surface.

The results of this work demonstrate that our proposed modular panel design robustly integrates a low-cost sensor to reliably measure distributed interaction forces in wearable devices. This sensorization approach can be used in a variety of interfaces and applications to monitor and control distributed loads. The measurements from our sensors further elucidates the fine effects of interaction between humans and wearable robots. This information is crucial to ensure safe, ergonomic, and effective human–robot interaction.

## 2. Sensing Solution Design

**Figure 2:**
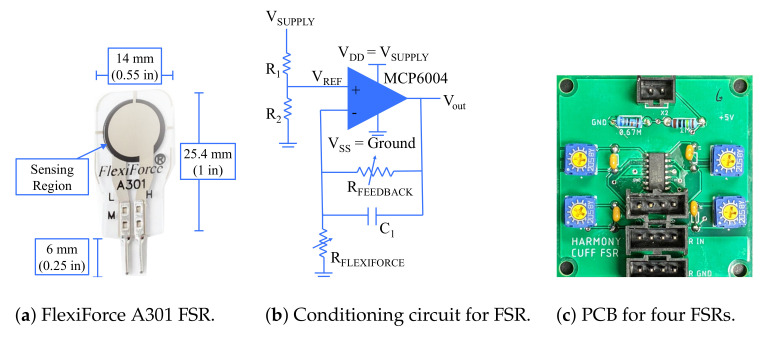
The force-sensing resistors (FSRs) are made from piezoresistive material that correlates the force applied on the sensing surface to the resistance of the sensor in an electrical circuit. This circuit requires the use of an operational amplifier. A printed circuit board has been designed for the implementation of the circuit shown in Figure 2b, such that each printed circuit board (PCB) accommodates four FSRs.

The sensing solution presented in this paper uses FSRs developed by Tekscan (South Boston, MA, USA) (Figure 2a) [17]. These sensors are designed to have a small form factor and are printed onto thin, flexible plastic making them compact and versatile. However, the integration of FSRs is not trivial and is the first challenge addressed in this paper. In the following subsections, we describe in detail the electrical integration and calibration process used for each sensor, followed by the details of mechanical integration.

### 2.1. Electrical Integration

FSRs use piezoresistive material such that the resistance they offer in a circuit depends on the force applied on the sensor. Thus, the voltage drop across the resistor is used to measure the applied force. The integration guide [17] presents multiple circuit designs that may be used for sensing this voltage drop. The single source excitation circuit (Figure 2b) is selected over the dual source one as the range of forces measured at the interface are not expected to vary significantly during use. This also minimizes the number of components required for the setup. As suggested by Tekscan, an MCP6004 operational amplifier from Microchip Technology Inc. (Chandler, AZ, USA) is used along with a trimmer potentiometer to adjust the feedback resistance for each sensor. As each MCP6004 chip can interface with up to four FSRs, each PCB accommodates four circuits as shown in Figure 2c. The MCP6004 chip is surface mounted to the board and the remaining components are soldered using through-holes (Table 1). The FSRs are connected to the circuit as per Figure 2b, and the output from the operational amplifier is measured using the CompactRIO through LabVIEW 2020, both from National Instruments (Austin, TX, USA). The measured voltage is converted to the corresponding force applied across the FSR through a calibration process.

#### 2.1.1. Calibration Protocol

The circuit diagram presented in Figure 2b provides a voltage output to the CompactRIO which must be converted to a force value sensed at each FSR. As per the integration guide [17], the single source circuit should result in a linear relationship between the voltage measured across the FSR and the force applied at the surface. However, literature has shown that this is not necessarily true and a nonlinear relationship may be used to better estimate applied forces [18]. In this paper, we considered the following three relationships:

(1) $$F=c_{0}+c_{1}V,$$

(2) $$F=c_{0}+c_{1}V+c_{2}V^{2},$$

(3) $$F=c_{0}+c_{1}V^{c_{2}}.$$

Equation (1) assumes a linear relationship, Equation (2) assumes a quadratic relationship, and Equation (3) assumes an exponential relationship between the force across the FSR (*F*) and measured voltage (*V*). The optimal relationship is determined through the following calibration protocol.

**Figure 3:**
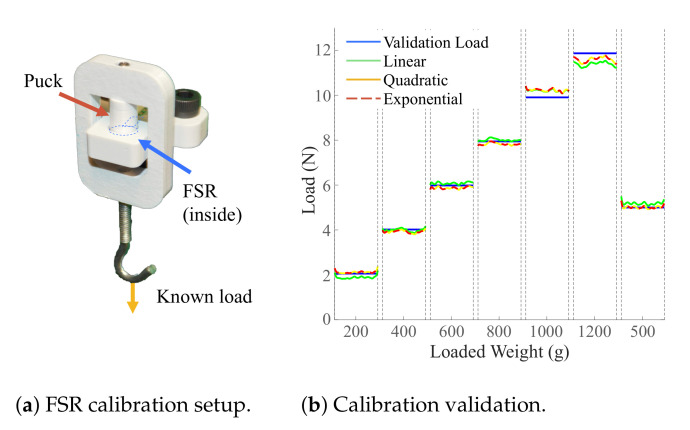
Calibration setup and results from a representative FSR (1 s of data under each loading condition). The linear relationship has the best performance across calibration tests across 24 sensors.

A sensor is placed in a calibration mount as shown in Figure 3a. A loading panel is interfaced with the sensor through an appropriately sized puck (Figure 3a), discussed in more detail in a later section. The loading panel is then loaded with six known loads of 1.96 N (200 g), 3.93 N (400 g), 5.88 N (600 g), 7.85 N (800 g), 9.81 N (1000 g), and 11.77 N (1200 g) followed by a seventh 4.9 N (500 g) load used for validation. The voltage across the sensor is measured at every load for a period of 5 s at a rate of 100 Hz. In order to avoid sensor noise from loading and unloading of the sensor, one second of data was used from the middle of the loading period. The signal is filtered with the “smooth” baseline function in MATLAB 2020 (Mathworks, MA, USA) which uses a moving average approach. The data points collected for the first six loads are used to solve for the coefficients in each of the Equations (1)–(3). Note that the weight of the calibration mount (0.095 N) is accounted for when solving for the voltage–force relationship. The resulting equations are used to predict the force values based on the measured voltage during loading with the seventh load (4.9 N, 500 g). These predictions are compared to the true load and a root mean square error (RMSE) value, averaged across 24 FSRs, is compared for the three relationships. The linear relationship is observed to have the lowest average RMSE of 0.34 N, followed by the quadratic relationship, 0.37 N, and the exponential relationship, 0.4 N. These results confirm that on average across different FSRs, the linear relationship suggested by the sensor integration guide [17] is optimal. A representative plot for one FSR comparing true loads and predicted loads in the calibration process is shown in Figure 3b.

#### 2.1.2. Data Collection

The two coefficients of the linear voltage–force relationship are computed and stored independently for each FSR prior to experimentation. Data from other sensors, including a six-axis load cell, are also collected for the experiments discussed in this paper. In order to compare the loads measured by the FSRs and other sensors, a time matching process is employed. The secondary sensor is loaded through a specific FSR to provide spikes in the data. These spikes are used to time match the recorded data in postprocessing. Next, the resulting load signal is filtered using the MATLAB 2020 (Mathworks, MA, USA) “smooth” baseline function. Finally, each FSR’s measured voltage values is converted to the corresponding loads by using the relationship derived during calibration.

### 2.2. Mechanical Integration

The overall mechanical design of the sensorized cuff presented in this section ensures that all loads at the human–robot interface surface will be captured by the distributed array of FSRs. We implement a panel design that individually interfaces with each sensor, and the interface surface for the full upper-arm cuff is discretized with 24 panels. Ergonomic design choices are also considered to ensure comfort while using the sensorized cuff given upper-arm geometry and soft tissue compliance. The mechanical design for the panel and sensorized cuff is performed in SOLIDWORKS 2019 by Dassault Systèmes SolidWorks Corporation (Waltham, MA, USA).

#### 2.2.1. Single Panel Design

**Figure 4:**
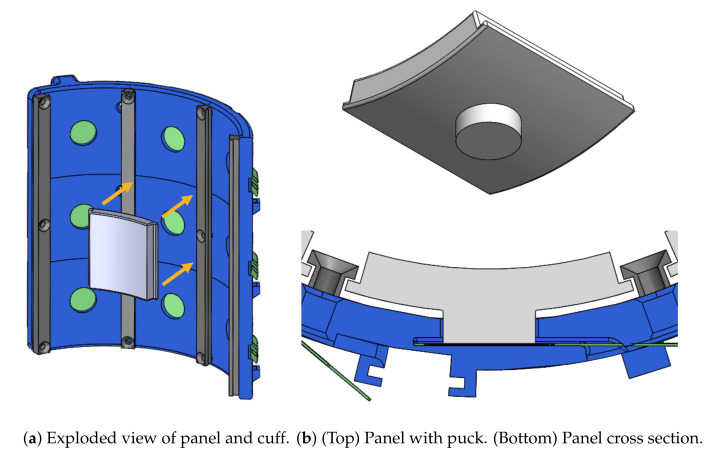
Integration of a single panel (white) installed in the sensorized cuff (blue) shows how it interfaces with the FSR (green) through a puck design. The divider rails (gray) keep the panels from falling out.

The design for a single panel is shown in Figure 4a. The goal of the panel design is to isolate loading normal to the human–robot interface surface and transmit it to the FSR without interference from loads in other directions such as shear. Furthermore, interaction forces from the contact surface should be entirely transmitted through the panels ensuring that information about distributed loading is not lost.

The panel design shown in Figure 4b uses a “puck” mechanism to transfer loads onto the FSR’s active sensing surface. This approach is recommended by Tekscan’s integration guide [17], especially when the load area is larger than the sensor’s active area. The puck acts as a load concentrator and ensures that the interaction forces from the top of the panel are fully transmitted to the FSR. The sensorized cuff includes a cylindrical channel which positions the puck directly on the FSR’s active sensing area and keeps the puck aligned normal to the sensing surface (Figure 2). In addition to capturing the entirety of normal forces from the panel surface, this design improves the repeatability of sensor data by transferring loads in a controlled manner and protects against sensor damage from point loads at high pressure. Divider rails on each side of the panel prevent it from falling out and restrain rotation around the axis of the puck. However, the rails are designed to not interfere with the panel in the direction of loading normal to the FSR and are never in contact with the human skin (Figure 4b).

The components within the panel design can be easily interchanged, making it ideal for a distributed sensing interface intended for research. For example, the white panels shown in Figure 4 can be switched out to test different interface padding as is done in Section 4.3. They can also be changed to experiment with different panel interface shapes (i.e., flat vs. curved) or different panel thicknesses that would change the effective inner diameter of the overall cuff. Similarly, the FSR integration is also interchangeable, allowing the sensors to be switched out if different FSRs are required. Thus, the panel design enables a variety of research applications with the same core hardware, saving time and effort that would be spent on extensive prototyping and fabrication.

#### 2.2.2. Cuff Design and Sensor Distribution

**Figure 5:**
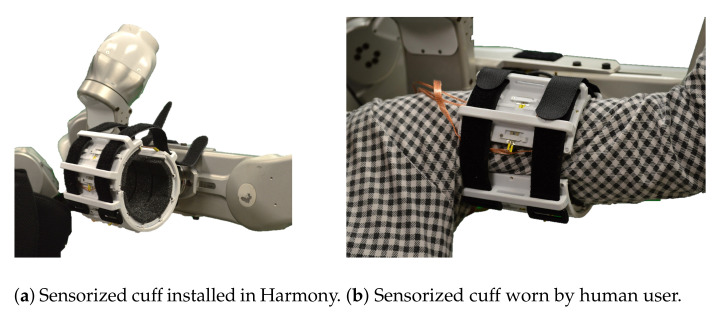
The sensorized upper-arm cuff is used with the upper-body Harmony exoskeleton and a human user to explore potential applications of this work.

**Figure 6:**
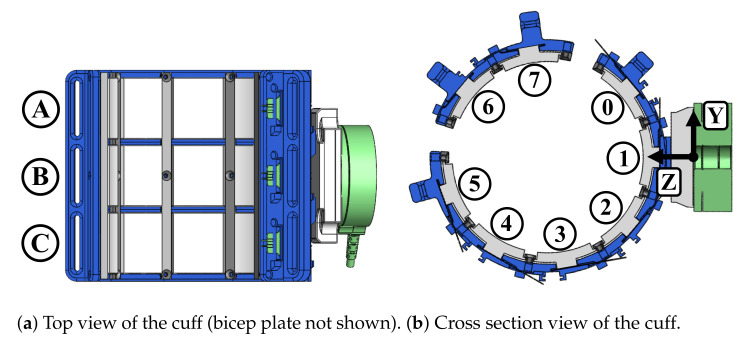
Sensor placement on the cuff is distributed across three rows and eight columns. Foam padding is not shown in these images. The orientation of the six-axis load cell can be seen in Figure 6b.

The extension of the sensing panel to a pHRI interface is demonstrated through an upper-arm cuff used to interface between a human wearer and the Harmony exoskeleton. The overall cuff design is shown in Figure 1. The main base of the cuff is accompanied by a bicep plate which is tightened by up to three velcro straps across the front of the arm (Figure 5). There are a total of 24 FSRs on the sensorized cuff with 18 on the base cuff and 6 on the bicep plate. The naming convention for sensor placement across three rows and eight columns, shown in Figure 6, designates the rows as A–C, with A closest to the shoulder and C closest to the elbow. The columns are designated 0–7, where 0 is the first column on the inside of the cuff and numbering proceeds clockwise. Columns 0–5 are on the main base of the cuff and columns 6 and 7 are on the bicep plate. There is 40 degrees of spacing between columns 0 through 5, and between 6 and 7. In order to allow for tightening of the straps around the bicep plate, columns 5 and 6, and 7 and 0 are separated by an additional 20 degree gap. Loading interference across panels is prevented by divider rails between columns and a gap between rows.

The length of the sensorized cuff is 94 mm and the inner diameter is 80 mm as measured from the surface of the panels. Additionally, the cuff is implemented with foam pads that result in an effective inner diameter of 74 mm. The standard foam used is 6 mm thick polyurethane (PU) intended for cushioning applications. The overall dimensions are sized to fit the 25th percentile value for female arm circumference [19] and can be scaled for larger arm sizes. Except for fasteners, all parts on the sensorized cuff were 3D printed with polyactic acid (PLA) filament. This manufacturing process lends itself to maintaining low-cost and allows for easy open-access use.

#### 2.2.3. Design for Ergonomics and Modularity

The comfort and ease of use of the sensorized cuff are prioritized during the design process to ensure applicability for studying ergonomics in wearable devices. The structure of the bicep plate and accompanying straps (seen in Figure 1d) accommodates some variability in arm sizes. Moreover, the use of multiple straps allows for adjustment to slight changes in arm diameter along the length of the arm for a single subject (i.e., upper-arm diameter is bigger close to the shoulder and smaller closer to the elbow). The integration of foam pads on the panels adds comfort for the user, and modularity of the panels can also help researchers easily switch between padding materials and study the trade-off between user comfort and interaction quality. The bicep plate design also leaves an opening in the main base of the cuff for easy donning and doffing. A quick release plate allows the user to wear the cuff while it is detached from the robot, further easing the donning process.

Our design approach first considered a single sensing panel and then expanded it to a sensorized cuff interface. In this sense, each panel is a modular sensing unit with standard electrical and mechanical design features. This modularity allows the researcher to only use certain sensing panels at a time if necessary. More importantly, the modular panel design enables scalability of the solution to different interface locations on the human body by implementation through an array of sensors. Thus, extension of the modular panel design can be used to explore a variety of pHRI interfaces without the need for an entirely new design process in each instance.

## 3. Validation of Sensing Solution Design

The sensorized cuff design is first evaluated with a validation testbed to confirm that loads applied on the distributed array of panels are correctly transmitted and observed in FSR measurements. Minimal error during these experiments validates that not only is the design effective in transferring normal loads across a single panel but also that simultaneous loading across multiple panels is correctly captured by the FSRs and is not lost during transmission.

### 3.1. Validation Experiment Testbed Setup

**Figure 7:**
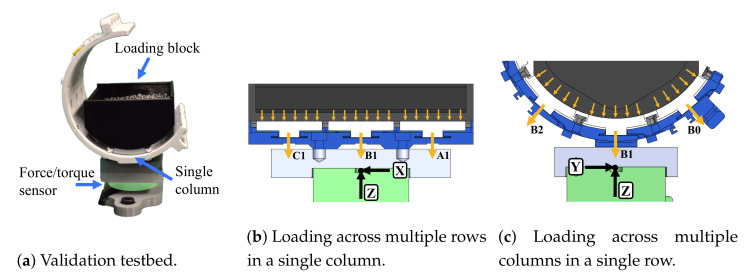
The validation testbed with the sensorized cuff mounted on a six-axis force/torque sensor (green). Distributed forces are applied with the loading block (black).

The validation testbed setup is shown in Figure 7. The sensorized cuff is mounted to a mechanical breadboard through a six-axis force/torque sensor (Robotous RFT40-SA01). A loading block sits on the inside of the cuff and applies weight to the panel surfaces based on the amount of lead pellets stored inside. Three weight levels are used for each experiment case during validation: 2.5 N, 5.0 N, and 7.5 N. Loads are transmitted to FSRs through the panel pucks and measured at the single-point six-axis force/torque sensor which serves as the ground truth.

**Figure 8:**
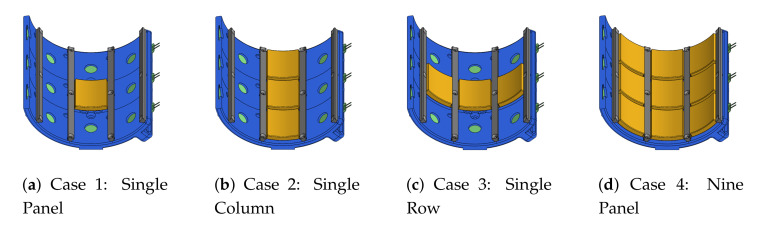
Four different loading cases are used to validate force transmission from panel surfaces to FSRs in the sensorized cuff. Only the panels highlighted for a given case are loaded in that test.

Columns 0, 1, and 2 are considered during the validation experiments, and four loading cases are defined to fully validate the design. The panels which are loaded during each case are highlighted in Figure 8, and all other panels are removed during the corresponding experiment. In the first case, only a single panel (B1) is loaded to validate the puck design by itself. In the second case, three panels in a single column (1) are loaded to validate that forces can be distributed across multiple rows. In the third case, three panels in a single row (B) are loaded to validate that the dividers do not interfere with force distribution across multiple columns. In the fourth case, all nine panels in rows A, B, and C and columns 0, 1, and 2 are loaded to validate the array of distributed sensors. This process is conducted first with panels alone and then repeated with standard polyurethane (PU) foam padding applied to the panel surfaces as it would be during use with a human subject.

### 3.2. Results from Validation Experiments

For each loading case and at each load level, a vector sum analysis is performed given the relative orientation of each FSR. The individual load observed at each FSR is converted to the vector components in the reference frame of the six-axis load cell (Figure 7). This is specifically relevant when considering the angle of FSRs around the circumference of the sensorized cuff in cases 3 and 4. Given that FSRs are spaced 40 degrees from each other across columns, an example calculation for the z-component from the three sensors shown in Figure 7c is given in Equation (4).

(4) $$F_{z}=-F_{B0}cos\left(40^{\circ }\right)-F_{B1}cos\left(0^{\circ }\right)-F_{B2}cos\left(-40^{\circ }\right)$$

**Figure 9:**
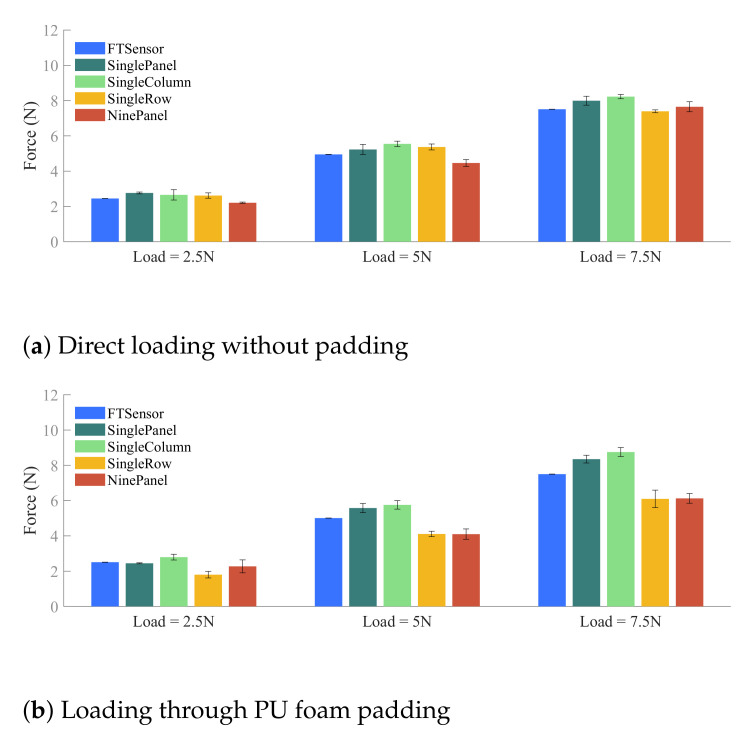
Results comparing the vertical loads measured by the force/torque sensor against the total sum of loads measured by the FSRs (in the vertical direction) for four loading cases: Single Panel, Single Column, Single Row, and Nine Panel, at three load levels. The error bars represent standard error across three repetitions. The error bars represent standard error.

The results of vector sums in the z-direction, which is the vertical axis directly loaded during validation experiments, are shown in Figure 9 for two interface surfaces: direct loading on the sensing panel and loading through PU foam padding. The first bar (blue) at each load level indicates the ground truth value as measured by the six-axis load cell at the base of the sensorized cuff. The other four bars correspond to the four validation experiment cases and represent the sum of forces in the z-direction across all panels.

### 3.3. Discussion of Sensorized Cuff Validation Results

The vector sum analysis shows good agreement between load cell measurement and FSR data in all validation cases. The mean predictions, also reported in Table 2, for the experiments conducted through direct loading of the panels are within 0.75 N of the known loads used for the experiments. The standard deviations remain within the window of calibration error seen in Section 2.1.1. The small value of standard deviations suggests the measurement of data is repeatable and robust to small fluctuations in load distribution. Note that in the cases where loading occurred across multiple columns, the resultant loads in the Y direction (Figure 8c,d) are expected to be close to but not exactly zero. The measured errors are consistent with those seen in the Z direction, and the largest resultant Y direction load is observed to be 0.61 N, averaged across the three repetitions of the third loading configuration in the direct loading case for the 7.5 N load.

To contextualize the errors observed in loading (Figure 9 and Table 2), we consider pressure pain threshold (PPT) which is a clinical metric used to define the minimum force which induces pain. While PPT measures vary depending on many factors, the minimum upper-arm PPT based on the work in [20] is 120 kPa. Accounting for the contact surface of a single panel, this indicates that errors below 1 N are the equivalent of less than 1.66 kPa, or 1.4% of the upper-arm PPT, confirming that the accuracy is sufficient for measuring distributed interaction forces.

In comparing the cases where panels were loaded directly versus through a layer of PU foam padding, we observed higher variability in the second condition. This discrepancy may be attributed to the way in which the foam was cut and applied to the panels. During experimentation, it was observed that the foam did interact across different panels as well as with other parts of the cuff such as the rails. This led to unintended interference along the columns of the cuff, coupling of loads across the rows, and offloading onto the cuff body. The exaggerated errors in the PU padding case are still close to or under 1 N.

## 4. Applications

Three potential applications of the sensorized cuff were explored to highlight how it may be used in physical human–robot interaction research. These uses extend beyond the upper-arm interface and can be studied at various attachments to the human body by using the modular design. All preliminary testing for this section was conducted in the context of the left arm of one human subject interfacing with an upper-arm attachment cuff. The first application considers the effect of strap tension on distributed interface loads. In the second and third applications, the left arm module of the upper-body Harmony exoskeleton [6] is used with the sensorized cuff during testing. In the second application, we observe interface loading during active user movement and robot-assisted passive user movement for the same motion. The third application considers differences that stem from the stiffness properties of two types of foam used as interface padding. The experiments and results described in this section are part of a pilot study conducted with only one subject. Thus, no generalizing conclusions are made regarding the effect of movement or interaction on the subject. The following discussion is aimed at demonstrating the many applications of our sensing solution.

### 4.1. Interface Loads at Different Strap Tensions

**Figure 10:**
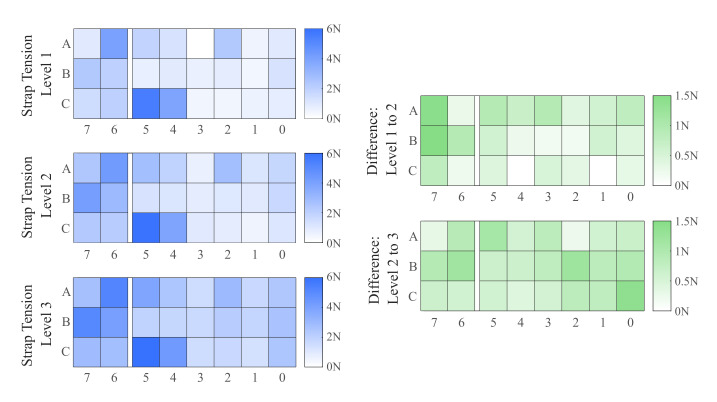
Heatmaps show distributed interface forces in Newtons across rows (vertical axis) and columns (horizontal axis). Note that columns 6 and 7 are on the bicep plate which is separate from the main body of the cuff. (Left) Distributed interface forces measured from FSR data at three strap tension levels where the third level is the tightest. (Right) Difference in distributed forces from the first level to the second level and from the second level to the third level.

In the first application, we consider how interface loads change at different strap tensions. While many wearable device attachments utilize straps, there is no consensus on predetermined strap tension when balancing functionality and user comfort. This is exacerbated by the difficulty of reliably measuring strap tension for multiple uses with the same subject and across different subjects. Moreover, the effect of changing strap tension is rarely detected in measurements from six-axis load cells at the attachment interface. During testing of this application, the user was instructed to self-tighten the sensorized cuff straps to a level that felt comfortable to them. This location was marked on each strap as the baseline strap tension level. Two more marks were made on each strap, spaced out by 10 mm in either direction, that indicated two additional levels of strap tension, one with higher strap tension than the baseline and one with lower strap tension. At each level of strap tension, the user rested their arm horizontally in front of them while their hand was supported by a fixture to minimize effects from muscle contraction. Data from FSRs were recorded for 10 s and compared at each strap tension level. The heatmaps in Figure 10 show the distribution of loading across the interface at different levels of strap tension as well as how the distribution of loads is spread out across the cuff.

Loading is observed to be the highest in column 5, specifically at panel C5, located at the edge of the main cuff (Figure 6b). This corresponds with earlier studies which have modeled and observed higher loading at the edge of pHRI interfaces [12,21]. As the strap tension is increased, better distribution of loading is observed in the central columns of the cuff. The heatmaps on the right of Figure 10, showing the changes in loading going from lower to higher tension, reveal that additional load is taken on primarily by the bicep plate (going from tension level 1 to level 2) and the inside edge at column 0 (going from tension level 2 to tension level 3). This further illustrates that much of the loading is concentrated near the strap tightening mechanism. The central areas of the main cuff such as columns 1–4 see less loading, largely due to the rigid nature of the cuff. Future work with sensorization of semi-rigid and soft interfaces will elucidate whether such mechanisms will be more ergonomic by spreading out interface loads.

### 4.2. Observations during Active and Passive Movement

**Figure 11:**
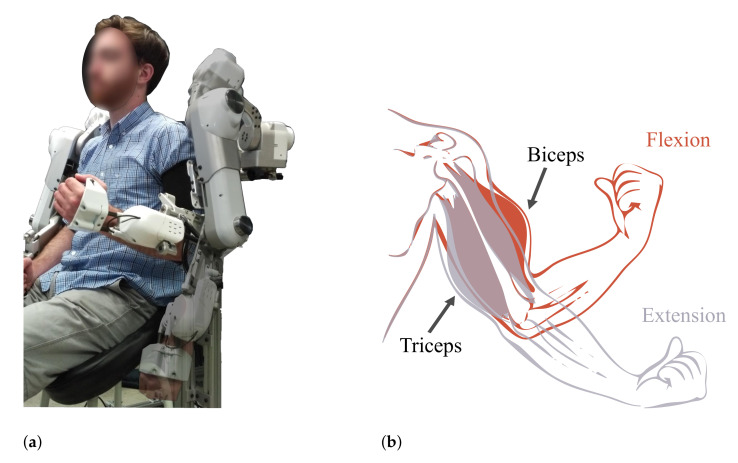
(**a**) Movement performed by the subject starts at full elbow extension (transparent), going to full flexion of the elbow (opaque) and back to full extension at the end of a single repetition. (**b**) Expected deformation of the arm’s muscles in response to the performed movements. Note that panel (**a**) is only a demonstration of the motion and not a representation of the experiment itself. (**a**) Elbow flexion movement in the Harmony exoskeleton. (**b**) Muscle contraction during elbow flexion and extension [22].

The second application uses the upper-body Harmony exoskeleton to make observations about interface loading during active human movement and robot-assisted passive human movement for the same motion. This demonstration is an example of how the sensorized cuff can be used to study user comfort, safety, and effort contribution during different modes of operation, especially for rehabilitation and assistive applications intended for sensitive populations. The sensorized cuff is donned at the baseline strap tension level deemed comfortable, and the user attaches into the left arm module of Harmony. The movement selected for this experiment is elbow flexion/extension (Figure 11a) because it minimizes motion of the upper-arm and focuses on the effects of passive versus active movement. During the active movement case, the exoskeleton compensates for the gravitational force acting on it. The human user starts with the elbow extended and actively performs five repetitions of elbow flexion/extension. A physical landmark indicates the hand position corresponding to the end of the flexion movement, and a metronome is used to control the frequency of motion. During the passive movement case, the user is relaxed and the exoskeleton performs the same elbow flexion/extension motion.

**Figure 12:**
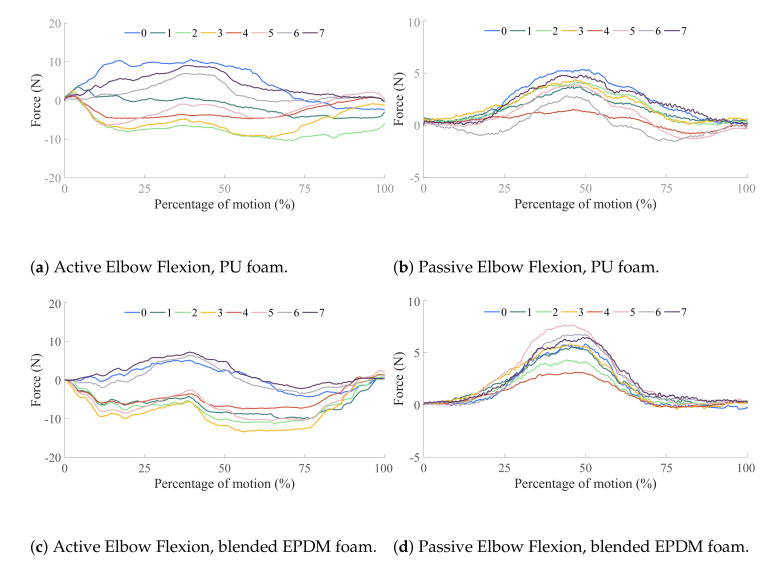
Results from the elbow flexion–extension movements performed by the subject while wearing the Harmony exoskeleton. Active movements refer to the subject performing the elbow flexion–extension while the robot follows in the gravity-compensation mode (accounting only for the robot’s own weight). Passive movements refer to the robot performing the movement while the subject follows passively. The elbow is fully extended at the beginning and end of each plot and fully flexed in the middle. The two foams—PU and blended EPDM—are shown in Figure 13.

Results from this experiment are shown in Figure 12a for the active movement and Figure 12b for the passive movement. These figures plot the sum of the three FSR loads in each column to observe how loading is distributed radially across the sensorized cuff. Overall, the active movement sees higher interface loading than the passive movement primarily due to voluntary muscle contraction from the human user. All columns in the passive movement case show the cuff interface being loaded in the same direction, indicating that this is the result of the Harmony exoskeleton pushing the upper-arm in a specific direction. On the other hand, the active movement case shows unique trends for different columns which are driven by the nature of muscle activations. As the elbow is flexed (at around 50% of the movement), columns 0, 6, and 7, located around the bicep muscle, see higher loads. Simultaneously, the columns corresponding to the triceps muscle, 2, 3, and 4, experience an unloading of forces. These results are in keeping with the expected change in arm shape due to activation of biceps during flexion (Figure 11b). The converse effect, relaxation of biceps and activation of triceps is also observed at the ends of the movements, corresponding to the extension of the elbow.

### 4.3. Effects of Interface Padding

**Figure 13:**
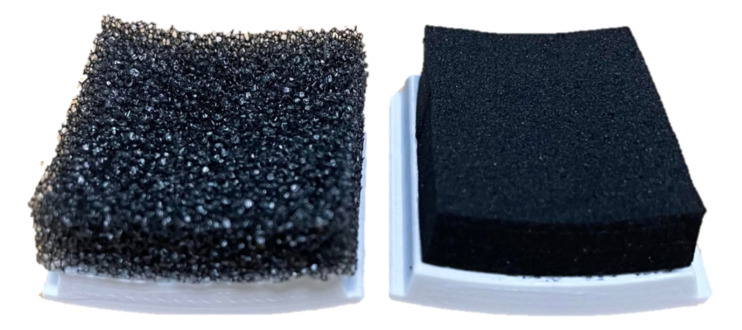
Two types of foam are compared in this paper: polyurethane or PU (left) and blended ethylene propylene diene monomer (EPDM) (right).

In the third application, the impact of different interface padding is studied (Figure 13), highlighting the insights that a sensorized interface can provide on how design choices influence ergonomics in wearable devices. The baseline foam used throughout this study is polyurethane, and is referred to as PU or PU foam. This is a standard foam used for cushioning applications. In this section, PU foam is compared with a blended ethylene propylene diene monomer (EPDM) foam that is roughly five times stiffer, referred to as EPDM or blended EPDM. The thickness used for both foams is 6 mm, and the interface padding is switched by changing the set of panels in the sensorized cuff which is enabled by the modularity in the design. The active and passive movement experiments from Section 4.2 are repeated with the blended EPDM foam and results are compared to the experiments with PU foam.

The effects of the two foams are studied by comparing Figure 12a and Figure 12b, which correspond to experiments with PU foam, to Figure 12c and Figure 12d, respectively, which correspond to experiments with blended EPDM foam. The trends in active and passive movements discussed in the previous section are also observed with the blended EPDM. Higher interface FSR loads are observed with the stiffer blended EPDM foam, especially for the passive movement and the unloading columns in the active movement. Comparison of FSR loads between polyurethane and blended EPDM shows that the former has more variability in trends across columns which might be caused by the more compliant nature of this cushioning foam. The higher variability corresponds to more distributed loading across the interface in the case of the softer PU foam compared to the stiffer blended EPDM foam. On the other hand, the stiffer blended EPDM causes more localized loading, which may be indicative of discomfort to the user. During experimentation, the subject also noted that the blended EPDM offered less variability during strap tightening. These experimental results demonstrate that the sensorized upper-arm cuff was able to differentiate between the effects of two distinct padding foams.

## 5. Discussion

The modular sensing panel proposed in this paper addresses two main challenges in distributed pHRI sensing: measurement of forces across the soft skin and a rigid interface, and implementation of an accessible sensing solution that allows it to be extended for various applications. Specifically, the design of the sensorized panel successfully integrates FSRs for the measurement of interaction forces across the human interface surface. The modular design is implemented in a sensorized upper-arm cuff with a grid of 24 panels. The paper presents rigorous experiments validating the sensing panel design and modularity as well as pilot studies with a single subject demonstrating the applications of the sensing solution.

The validation results demonstrate the efficacy of the design presented in this paper. The amplifier circuit and PCB design reduce noise and increase robustness of the sensor signal. The novel panel design reliably transfers loads from the human–robot interface to the FSR’s sensing region, and the design further ensures minimal coupling across adjacent sensor panels. The experiments validating the single panel design and its extension to a grid prove these sensors can be used to sense distributed loading at the pHRI interface. The integration of FSRs confirms their reliability beyond that suggested by the results of earlier works [10,15,23] and provides a low-cost option for evaluating ergonomics in wearable devices. The cost of a single sensing panel falls between $15 and $16. The overall cost of the sensorized cuff presented in this paper is thus less than $400. With a unit price of $13.83, FSRs constitute the main proportion of the cost of the overall cuff with 24 sensors, adding up to $331.80. The cost for other aspects such as PLA filament (<$10), foam padding (<$10), and circuitry (<$20) is minimal. While a direct price comparison with opto-electronic solutions is not feasible, as that technology is not available off-the-shelf, our sensorized cuff is much cheaper than state-of-the-art distributed sensing systems such as the ones from Tekscan [24] and Novel [25] which fall in the range of $25,000 to $35,000 with the majority of the cost attributed to the proprietary data acquisition electronics and software.

The modular panel design can also be used for other attachment shapes designed for various parts of the human body. In this paper, we demonstrate the use of 24 panels in a sensorized upper-arm cuff that is used in conjunction with the Harmony exoskeleton, but the sensing solution can also be scaled for different attachment locations on the human body. The modular panel design can be sized accordingly and adapted for the pHRI interface shape. In fact, the curvature of the panels does not need to be standardized throughout the surface and can be customized for specific locations on the interface (i.e., a lower back brace can have flat panels in the middle and curved panels on the edges where it wraps around the abdomen). All other aspects such as the mechanical design, electronic circuitry, and sensor calibration easily translate to distributed sensing solutions at different interfaces. This is in contrast to previous works [9,12,15,16], which approach the design and fabrication process while considering a specific pHRI interface as a whole instead of considering a modular sensing unit, leading to solutions that cannot be easily scaled or adapted for different attachment locations on the human body.

Distributed interface force sensing provides more in-depth information than conventional single-point six-axis load cells. The discussed applications confirm that a sensorized interface in active wearable devices such as exoskeletons can provide better information regarding user comfort and safety which is especially relevant in rehabilitation. Further applications lie in haptics where the fine nature of interaction forces is critical for understanding how the human perceives their environment [26]. Last, the sensorized upper-arm cuff provides meaningful information for the development of design paradigms [21,27] for optimizing ergonomics in wearable attachments.

Future work will focus on studying the aforementioned applications further in the upper-arm cuff and extending interface sensorization to other attachments in wearable devices. The experiments discussed in Section 4 form a pilot study with a single subject. We plan to further study the effects of interaction with a larger subject population towards deriving generalizable conclusions. Other applications we plan to explore include the estimation of assistive torque and active subject effort, as well as the potential for integrating FSRs in semi-rigid and soft attachments while maintaining reliable sensor measurements. Ultimately, the modular sensing panel and the demonstrated sensorization of interfaces lays the groundwork for the future of wearable devices designed to efficiently and ergonomically integrate with the human user.

## Figures and Tables

**Table 1:** Components used in the PCB design for sensing of the voltage drop across the FSRs.

Component Type	Name/Value
Operational Amplifier	MCP6004
Capacitance	15 pf
Power Source	5 V
Resistance 1	1 MΩ
Resistance 2	0.68 MΩ
Potentiometer	2 MΩ

**Table 2:** Load predictions across four loading cases, three levels of loading, and two types of surfaces.

	Direct Loading without Padding			Loading through PU Foam Padding		
	**Load = 2.5 N**	**Load = 5 N**	**Load = 7.5 N**	**Load = 2.5 N**	**Load = 5 N**	**Load = 7.5 N**
**Single Panel**	2.76 ± 0.09 N	5.23 ± 0.50 N	8.00 ± 0.44 N	2.45 ± 0.05 N	5.58 ± 0.45 N	8.35 ± 0.38 N
**Single Column**	2.66 ± 0.51 N	5.55 ± 0.26 N	8.23 ± 0.21 N	2.79 ± 0.28 N	5.76 ± 0.42 N	8.75 ± 0.74 N
**Single Row**	2.62 ± 0.26 N	5.37 ± 0.29 N	7.39 ± 0.14 N	1.80 ± 0.32 N	4.11 ± 0.27 N	6.10 ± 0.86 N
**Nine Panel**	2.21 ± 0.07 N	4.46 ± 0.34 N	7.65 ± 0.49 N	2.27 ± 0.64 N	4.10 ± 0.51 N	6.13 ± 0.48 N

## Abbreviations

The following abbreviations are used in this manuscript:

pHRI	Physical Human–Robot Interaction
FSR	Force Sensing Resistor
PCB	Printed Circuit Board
PU	Polyurethane
EPDM	Ethylene Propylene Diene Monomer
PLA	Polyactic Acid
PPT	Pressure Pain Threshold
